# Mechanical Behaviour of Umbrella-Shaped, Ni-Ti Memory Alloy Femoral Head Support Device during Implant Operation: A Finite Element Analysis Study

**DOI:** 10.1371/journal.pone.0100765

**Published:** 2014-06-24

**Authors:** Wei Yi, Qing Tian, Zhipeng Dai, Xiaohu Liu

**Affiliations:** 1 Department of Mechanics, Huazhong University of Science and Technology, Wuhan, Hubei, China; 2 Tongji Medical College, Huazhong University of Science and Technology, Wuhan, Hubei, China; 3 Tongji Medical College, Huazhong University of Science and Technology, Wuhan, Hubei, China; 4 Department of Mechanics, Huazhong University of Science and Technology, Wuhan, Hubei, China; University of Eastern Finland, Finland

## Abstract

A new instrument used for treating femoral head osteonecrosis was recently proposed: the umbrella-shaped, Ni-Ti memory femoral head support device. The device has an efficacy rate of 82.35%. Traditional radiographic study provides limited information about the mechanical behaviour of the support device during an implant operation. Thus, this study proposes a finite element analysis method, which includes a 3-step formal head model construction scheme and a unique material assignment strategy for evaluating mechanical behaviour during an implant operation. Four different scenarios with different constraints, initial positions and bone qualities are analyzed using the simulation method. The max radium of the implanted device was consistent with observation data, which confirms the accuracy of the proposed method. To ensure that the device does not unexpectedly open and puncture the femoral head, the constraint on the impact device should be strong. The initial position of sleeve should be in the middle to reduce the damage to the decompression channel. The operation may fail because of poor bone quality caused by severe osteoporosis. The proposed finite element analysis method has proven to be an accurate tool for studying the mechanical behaviour of umbrella-shaped, Ni-Ti memory alloy femoral head support device during an implant operation. The 3-step construct scheme can be implemented with any kind of bone structure meshed with multiple element types.

## Introduction

Osteonecrosis of the femoral head is a devastating disease that typically presents in young patients between the ages of thirty and forty [1]. If osteonecrosis is not effectively treated, many patients will experience femoral head collapse [2]. Hip replacement is not an optimal choice for the younger patients because of the validity of the artificial joint. Therefore, several minimally invasive surgery modalities [3] have been used to prevent the collapse of the femoral head. The following treatments, in the order they’ve been introduced, have been used in therapy: transtrochanteric osteotomy [4], core compression [5], Nonvascularized bone graft [6], vascularized bone graft [7], porous tantalum implant [8], Ni-Ti superelastic cage implant [3]. Of these treatments, those that used implant techniques were much more effective than the others [3]. This is because the structural stiffness of the implanted device provides a high level of support. However, all of these devices come with certain limitations, including a support area that is too small. Recently, an umbrella-shaped, Ni-Ti alloy femoral head support device [9] was proposed to treat the early stage osteonecrosis of the femoral head. Based on 17 devices implanted in 10 patients, the device showed an 82.35% efficacy rate. As reported, the support area was much larger than the traditional implant treatment. Note, however, that there are also risks associated with this operation. The biomechanical mechanism was not entirely clear, and the operation process has not yet been perfected.

Ni-Ti shape memory alloy, as a functional metal material, has many advantages including a remarkable resistance to wear and corrosion, good biocompatibility, and other special mechanical characters: SME (shape memory effect) [10], PE (pseudo elastic) and pseudo plastic [11]. These distinct characteristics make the shape memory alloy very useful in bone surgery instrument design [12]. The many studies on the biomechanical characteristics of the Ni-Ti SMA (shape memory alloy) have increased its used in orthopedics. From the work of Auricchio [13], Lsdyna provides a linearized macroscopic phenomenological model for SMA spans, which is widely used for SMA simulation. Although this model did not include permanent strain, PE was still in effect.

The major focus on Ni-Ti surgery instruments has been in clinical practice, with few computational studies conducted on the instruments. This is particularly true for the umbrella-shaped formal head support device, which has yet to be computationally researched. The FEA (finite element analysis) can serve as the deformation field, strain field and stress field of the femoral head and support device, which is almost impossible to determine in a radiological imaging study. More importantly, the computational simulation study can predict the whole process without actually performing the surgery. Because the bone is damaged inside the femoral head, and a traditional static analysis is unable to address issues related to element failure, the explicit integration method is used.

The aim of present study is to propose a finite element analysis method based on Lsdyna to simulate the mechanical behaviour of the Ni-Ti umbrella-shaped femoral head support device during the implant process. This method includes 1) a 3-step model construction and particular material assignment strategy for the femoral head in FEA pre-processing and 2) a dynamic relaxation analysis (the normal step) in which the stress initialization file is derived from a pre-analysis (the pre-step) of the support device.

We attempted to illustrate the expanding process within the femoral head during surgery, and review the accuracy of the simulation method by comparing the literature data with the simulation. Two different cases of sleeve initial positions were simulated. The results suggested that the open shape might be similar, but different kinds of damage were found inside the femoral neck. Finally, we conducted a simulation of the same patient with moderate osteoporosis. The results show that moderate osteoporosis might lead to surgery failure because the femoral head was not strong enough to resist the expending of the implant device. Since the finite element simulation method was effective and accurate, it might also be used as a rehearsal for selecting both the best implant location and implant device size for patients with varying bone qualities. Equation Chapter 1 Section 1.

## Materials and Methods

The FEA study is based on a 3-step model construction scheme, outlined below. Initially, the model is digitized based on a CT (Computed Tomography) scan experiment. Then, the geometric model is created with Mimics and Geomagic Studio. The finite element is then discretized and a special material assignment strategy is carried out. Finally, an analyses is conducted during which the pre-step simulates the reshape phase under 0°C in vitro and the normal step simulates the opening phase under 50°C in vivo. The Boundary conditions and loading are explained in a following subsection.

### CT Scan Experiment

The first step of the 3-step construction scheme was to digitize the geometric and bone quality information of the femoral head. An anonymous 45-year-old male patient with Stage I femoral head osteonecrosis was CT scanned every two millimeters from the femoral head to the lesser trochanter and then at five millimeter intervals along the femur bone. A Brilliance 128 iCT scanner (Philips) was used to scan the patient. The CT slice image data were saved and exported in DICOM (Digital Imaging and Communications in Medicine) format.

**Figure 1:**
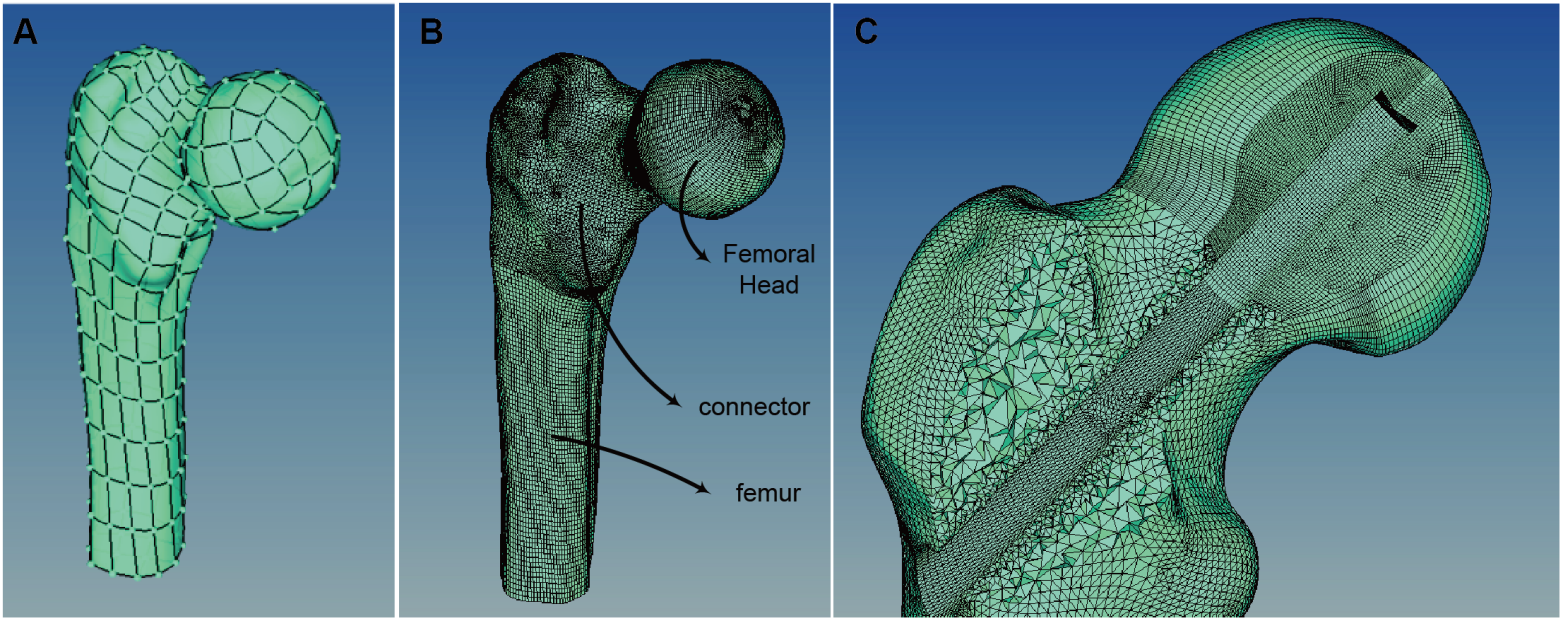
Digitized femur. (A) Geometric Model; (B) FE mesh model; (C) FE mesh model (local view).

The second step was to reconstruct a high quality 3D geometric model based on the digitized slice image data. The slice images were imported into Mimics software (Materialise, Leuven, Belgium) for the model reconstruction. Then, using Geomagic Studio V13.0 (Raindrop Geomagic, TrianglePark, NC) with a reverse modeling process, a fine geometric model was made (Fig. 1(a)).

### FEA Analysis

#### Mesh and material assignment of femoral head and femur

After the Geometric model was constructed from the CT images, an operation channel with a 9 mm outer diameter at the greater tuberosity to a 5 mm diameter under the subchondral bone along the femur neck was cut in the formal head. The last step of the construction scheme was element discrete.

In HyperMesh, we used the geometric model created during the pre-step to carry out the mesh process. The whole bone structure was divided into 3 parts: the femoral head, the femur, and part in-between. The first 2 parts were meshed with 8-node 1^st^ order hexahedron elements. Because the femoral head part was the main area of focus, the mesh with the element edge in the range of 0.28 ∼0.62 mm was much finer then the femur part. The 3^rd^ part was meshed with 4-node 1^st^ order tetrahedron. The element numbers, element types, and support device are shown in Table 1.

**Table 1:** Mesh Information.

Parts	femoral head	femur	connector	umbrella	sleeve	total
Number	113680	59080	139366	11904	5412	329442
Element nodes	8	8	4	8	8	4 & 8
Element type	hexahedron	hexahedron	tetrahedron	hexahedron	hexahedron	h & t

The low quality (2 mm resolution) of the CT scan data prevented a clear view of the internal femoral bone microstructure and distribution of the internal trabecular bone, but it provided enough information to establish a high quality 3D geometric model of the femur. The quality of the mesh depends only on the meshing skill when the geometric model is adequate. In other words, the low quality of the CT scan has no effect on the element quality when a high quality 3D geometric model can be made.

After the 3-step model construction, we assigned a material property for each element. Because of the limitations of the Mimics auto-assignment function (it is only available when the mesh is created with 4-node elements), an assignment strategy was used.

**Figure 2:**
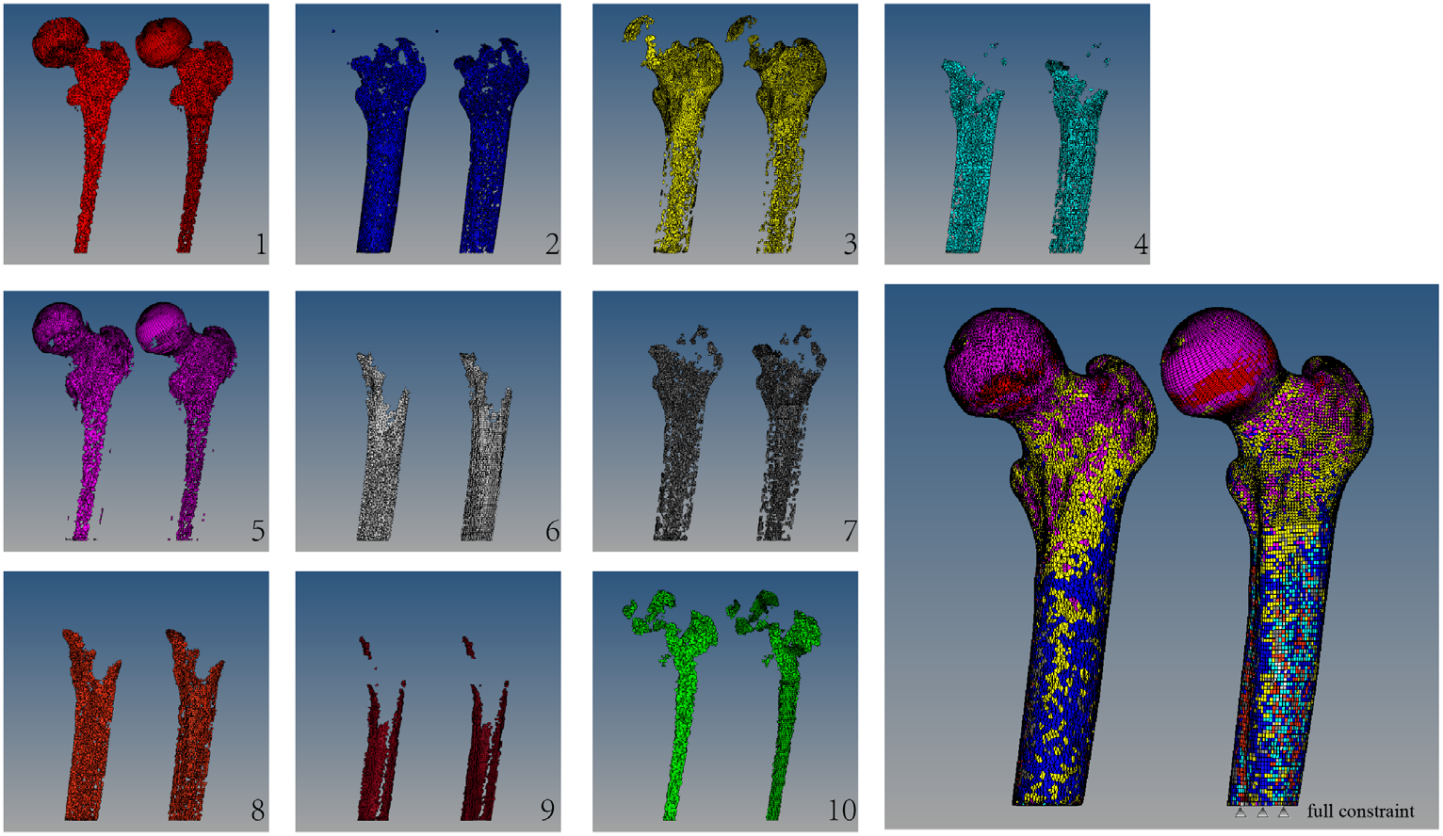
Bone material assignment results of 10 individual groups and the overall model. Constraint on the bone structure: In the normal step, a full constraint was applied at the bottom to eliminate rigid displacement of the whole structure.

Unable to use the Mimics auto-assignment function for the mixed-type mesh (mesh II), we generated a 4-node tetrahedron element mesh, named mesh I, and auto-assigned material in Mimics. The problem then presented is how to link Mesh I and II to each other. The detailed process for solving this problem is as follows:

Step 1: Generate an auto-assigned density for Mesh I in Mimics as 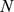 sets of elements. All of the sets are created based on the range of the apparent density which was evenly distributed and shown as the left side of each sub-graph in Fig. 2.
Step 2: Correspond Mesh II to Mesh I using the mass centroid coordination of each element. Since Mesh I is divided into 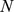 sets, Mesh II is also divided into 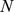 sets with the same apparent density as Mesh I. This can be seen in the right side of each sub-graph in Fig. 2.
Step 3: Based on the porosity-elastic module squared relation [14], 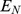 for set 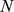 is determined along with the failure strain for all the bone elements [15].

It’s important to note that Mesh II is much finer than Mesh I. Hence, a cubic search bucket with a width 20 times the longest side of the current element 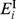 is created. In this bucket, we searched the corresponded element 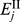 that the mass centroid of 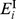 belonged to.

The 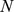 is set to 10, and the density, elastic module, failure strain and Poisson’s ratio of each set are shown in Table 2. The corresponding elements of each set in Mesh I and II are shown with the same color, side by side. (Mesh I is show on the left side, and mesh II on the right).

**Table 2:** Material parameters for 10 different sets of bone elements.

Set | Material Property	Density (mg/mm^3^)	Elastic module (Gpa)	Failurestrain	Poisson’s ratio
1 | 2	0.63(0.53)	0.80(0.56)	0.009	0.3
2 | 5	1.57(0.31)	5.00(3.5)		
3 | 4	1.26(1.05)	3.20(2.24)		
4 | 7	2.19(1.83)	9.80(6.68)		
5 | 3	0.94(0.79)	1.80(1.26)		
6 | 9	2.83(2.37)	16.20(11.34)		
7 | 6	1.88(1.57)	7.20(5.04)		
8 | 8	2.51(2.10)	12.80(8.96)		
9 | 10	3.14(2.63)	20.00(14.00)		
10 | 1	0.32(0.27)	0.20(0.14)		

In Mimics, a linear formula is used to translate the Hounsfield into the bone mass density field by the linear formula:
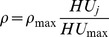
(1)Where, 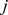 is the material property number. It is important to note that material property number and element set number are not necessarily the same, as shown in Table 2. 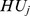 represents the 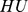 value of the material property 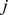, and can be calculated with formula (2):



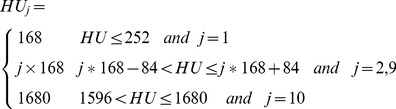
(2)The 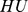 value range 168∼1680 was used to separate the muscle and soft tissue, while maintaining the best shape of the femur and formal head.

With 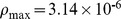 Kg/mm^3^, the bone mass density field is calculated. Then, the squared relation [14] is used to calculate the elastic module:
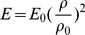
(3)Where 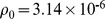 Kg/mm^3^, 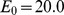 Gpa. Because of a lack of previous experiments conducted on actual femoral head bone material, the approximate squared relationship is a good and relatively simple choice. Moreover, it is often used for bone structure simulations [21].

The equivalent strain failure criterion was used to judge the failure of the bone element. When the equivalent strain reached 0.009, the bone element was damaged and removed from the calculation. This element deletion will damage the original contact surface area, which forms on the bone element’s surface. Thus, all the contacts involving bone are set to an eroding-type contact, in which the contact surface can be updated whenever the original contact surface was damaged.

When the patient suffered from severe osteoporosis, a 16.34% bone mass loss was assumed and the elastic module was 30% less than normal (Table 2). The values in and out of the brackets correspond respectively to the osteoporosis bone and normal bone.

### Umbrella-shaped Femoral Head Support Device

**Figure 3:**
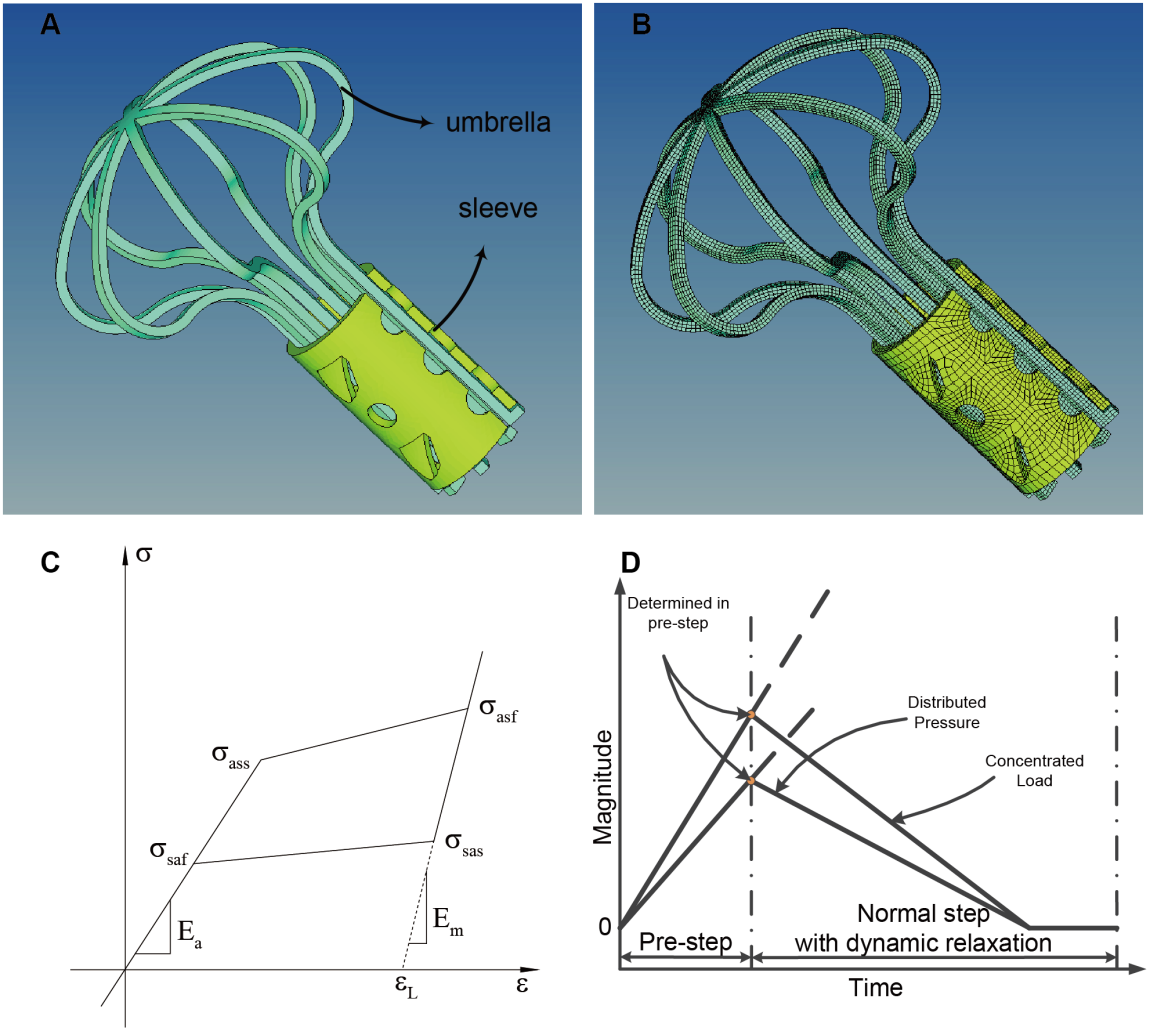
Implant devices and analysis setting in Lsdyna. (A) Umbrella-shaped femoral head support device geometric model; (B) Umbrella-shaped femoral head support device FE mesh model; (C) Material model in Lsdyna; (D) User-defined load curve in pre-step and normal step in: Lsdyna.

The geometric model of the Ni-Ti umbrella-shaped femoral head support device at 50°C is shown in Fig. 3(a). The implant device contains two parts: the umbrella and the sleeve, marked in green and yellow, respectively. The outer diameter of the sleeve is 12 mm. The umbrella height is 50 mm; the outer diameter is 36.25 mm; and the width of the umbrella arm is 1.2 mm. The thickness is 1 mm in both parts. The umbrella and sleeve are all meshed with 8-node hexahedron elements. There are at least 3 layers of elements width-wise. The element numbers of the umbrella and sleeve are shown in Table 1.

### Ni-Ti SMA Constitutive Relation

There are three kinds of constitutive relations that can be used to describe the shape of the memory alloy: a microscopic thermodynamic model, macroscopic phenomenological model [16], and multivariant micromechanical model [17]. The microscopic thermodynamic model is useful for determining the nucleation, interfacial movement, growth of martensite, and other microscopic mechanisms of a material. Using the microscopic thermodynamic model, the macroscopic constitutive relation in the Multi-scale model is determined. Although the physical meaning of these two methods is clear, they remain difficult to use in practical engineering. The macroscopic phenomenological model [18], [19], [20] has developed rapidly in concert with the enrichment of experimental results. However the phenomenological constitutive model equation and the phase transition thermodynamics equations are nonlinear. In other words, it’s easy to not use these equations as they require a many resources and demand a great deal of time to solve.

**Figure 4:**
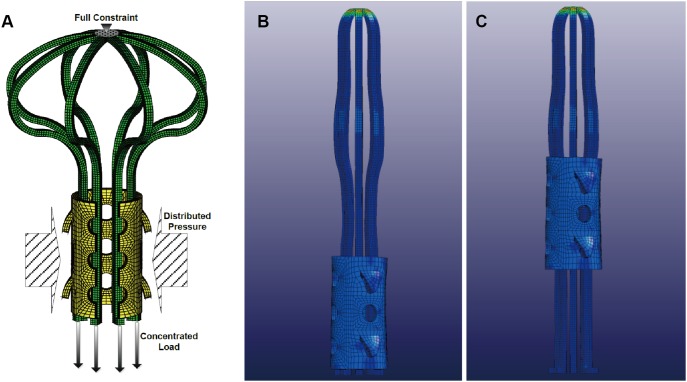
Constraint and load on implant devices and two different positions of the sleeve. (A) constraint and load on implant devices in pre-step and normal step:a full constraint at the top of the umbrella, concentrated load on the bottom of the umbrella, distributed pressure on the sleeve, the loads increase with time in the pre-step and decrease with time in the normal step; (B) Position I: sleeve at the bottom; (C) Position II: sleeve in the middle.

As previously mentioned, Lsdyna provides a simplified macroscopic phenomenological model through which the phase-changing process is made linear (Fig. 4(a)). Where 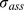 and 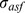 are the start and final stress values for the forward phase transformation, respectively. 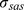 and 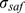 are the start and final stress value for the reverse phase transformation, respectively. When the ambient temperature of 50°C was higher than 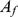, which is the Austenite Finish temperature (material parameter which is 37 °C), the stress-train curve of the SMA under an isothermal environment is determined (Fig. 3(c)). The mechanical load placed upon the alloy produces a stress on the martensite phase transformation; when the stress reaches 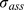 the alloy starts to transform from the austenite phase to the martensite phase. When the stress reaches 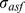 the forward transformation is complete. This type of phase transformation remains stable only when under a mechanical load. Even if unheated, when the mechanical load was lifted the inverse transformation occurred. When the stress decreases to 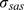, the inverse transformation to austenite begins; when the stress reaches 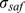, the inverse transformation occurred. When the stress decreased to 0, i.e. when the mechanical load was essentially cancelled, the SMA recovers to its original shape.

The material parameters are shown in Table 3. Temperatures 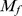, 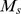, 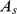, 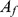
(representing Martensite Finish, Martensite Start, Austenite Start, and Austenite Finish, respectively) are set to 9°C, 18.4°C, 29.3°C,and 37°C. These temperatures are set in accordance with Brinskon [20] and Yu [9]. The transformation constants 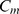 and 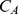, used to describe the relationship of the temperature and the critical stress to the induced transformation, are 0.008 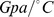 and 0.0138 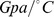, respectively. 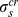 and 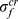 which represent the critical stress value below 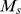 are 0.1 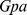 and 0.17 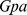, respectively. Equations for evaluating the four stress values used in Lsdyan from the material parameters mentioned above are:
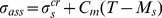
(4)

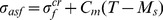
(5)

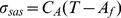
(6)

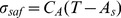
(7)


**Table 3:** SMA material parameters setting.

Density(mg/mm^3^)	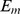 (Gpa)	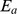 (Gpa)	Poisson’sRatio	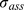 (Gpa)	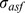 (Gpa)	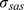 (Gpa)	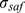 (Gpa)	_EPSL_	_ALPHA_
_6.3×10_^−6^	63.0	26.3	0.3	0.3528	0.4228	0.2856	0.1794	0.067	0.0

When the temperature was set to 50°C, the material parameters used in Lsdyna are obtained and shown in Table 3.

The EPSL refers to the maximum residual strain; and the ALPHA refers to the parameter measuring the differences between the material responses in for both the tension and compression.

### Constraint and Load

The simplified linearized pseudo elastic model does not include permanent strain. In other words, after unloading, there is no stable deformation. This remains true even if the deformation is larger than the maximum strain set to.067, as described in more detail above. To properly maintain the shape below 0°C, the constrained boundary and a tension load are applied on the umbrella. A pressure is also applied on the sleeve in the normal step, as seen in Fig. 4(a). Note that the constrain and load shown above are the same values used to calculate the shape of the support device under 0°C in the pre-step. The magnitudes of these loads, i.e. the inflection points in Fig. 3(d), are chosen so that the device matches the shape presented in previous study [9]. More specifically, the outer diameter of the umbrella is 8 mm and the outer diameter of the sleeve is 9 mm.

After the reshape phase under 0°C (the pre-step), the bone structure with a full constraint at the bottom was added to the simulation (the normal step). When the distributed pressure and concentrated load began decreasing, the umbrella and sleeve began to recover to their original shape, leading to bone element failure. By the end of the simulation, the artificial load on the device has decreased to zero, and the final shape is obtained. The user-defined load curves of the distributed pressure and concentrated load are shown in Fig. 3(d). Two different phases–the reshape phase (pre-step phase) and open phase (normal step phase)–are included. The inflection points are determined in the pre-step phase with multiple computations.

Although the surgery process has been comprehensively described, the optimized initial position of the support device has not been determined. Likewise, there are problems remaining that relate to the damage inside the femoral head as well as variability of bone quality as seen in different patients. Thus, in the normal step phase, 4 different scenarios are simulated (Table 4).

**Table 4:** Information of four different scenarios.

Scenario	Initial Position		Bone Quality		Constraint and Load	
	I	II	Normal	Osteoporosis	Yes	No
1	√		√			√
2	√		√		√	
3	√			√	√	
4		√	√		√	

Scenario 1 is the only one scenario without a constraint: the umbrella and sleeve can move freely, no load has been applied the umbrella and the sleeve, and the initial position I is used (Fig. 4(b)). In Scenario 2, we constrain the umbrella at the top; the concentrated load and distributed pressure are slowly released. Scenario 3 is almost the same as scenario 2, except that the bone material is different. Scenario 4 is the only scenario that uses the initial position II (Fig. 4(c)); otherwise, the conditions are identical to Scenario 2.

In all 4 scenarios, the contacts are set for the umbrella, the sleeve and formal head with each other. Two self-contacts are set for the umbrella and sleeve. More specifically, eroding-type contacts are implemented when formal bone is involved. When bone elements are deleted because of failure, a new contact surface is generated in the eroding-type contact.

## Results

**Figure 5:**
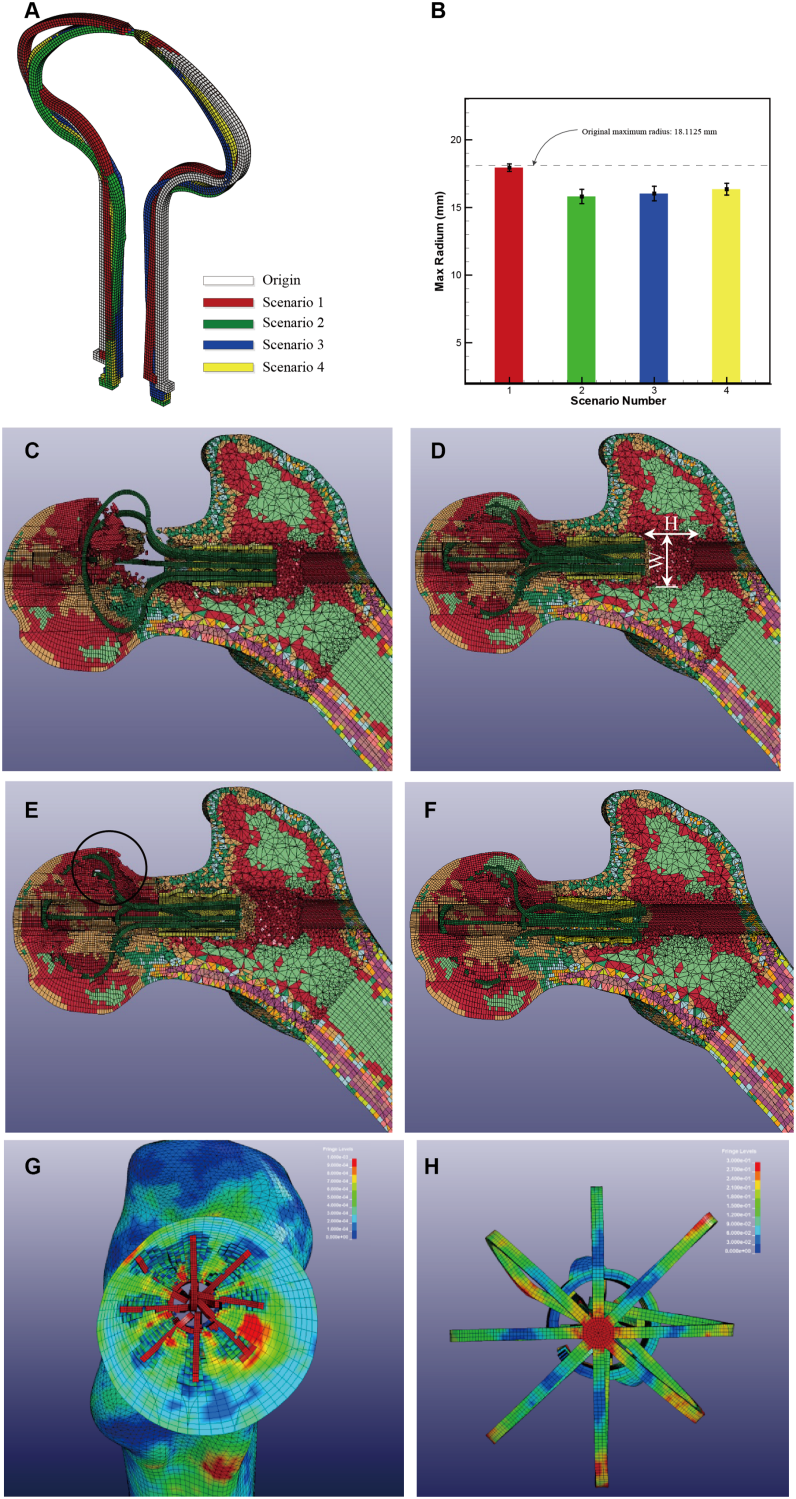
Analysis results. (A) Final shape of one typical pair of 4 opposite umbrella arm pairs in each scenario; (B) Statistical figure of maximum radius of each scenario; (C) scenario 1, without constraint; (D) scenario 2, initial position 1, Normal bone quality. H = 22.5 mm, W = 20 mm; (E) scenario 3, with osteoporosis, failure location marked with black circle line; (F) scenario 4, initial position 2; (G) Stress field distrubution inside femoral head in secnario 4; (H) Stress field distrubution of umberalle-shape device in secnario 4.

After reshaping under 0°C, the Ni-Ti alloy umbrella-shaped support device is implanted in the formal head. Hot saline (50°C) is poured into the formal head, and the support device begins to recover to its original shape. The primary focus of this research is to evaluate how the final shape of the support device functions in the human body. However, because of the resistance of the cancellous bone inside the formal head, the final shape is not the same as original shape, (Fig. 5(a)). In other words, the support device cannot totally recover its shape. Likewise, there is a compression deformation with respect to the original shape. Thus, the support device contains a potential energy expansion, and plays an active role in allowing the formal head to resist the pressure caused by normal bodily activities.

After the simulation, several conclusions were reached:

1. The top of the umbrella should be fully constrained to avoid the umbrella opening in an unexpected location (Fig. 5(c)). When the umbrella penetrates the formal head, the head suffers severe bone damage.
2. In Scenario 2, the final shape of the support device is similar to the original shape but with a decreased outer diameter. The outer diameter of the sleeve decreased, on average, from 12 mm to 11.26 mm (Fig. 5(b)). The maximum outer diameter of the umbrella decreased on average from 36.225 mm to 31.62 mm, which is quite close to previously observed data (32 mm) [9]. A typical pair of 4 opposite umbrella arm pairs for each scenario is shown in Fig. 5(a).
3. In Scenario 3, the operation failed, penetrating the femoral heads of the patients with severe osteoporosis. The failure position was near the bottom of the femoral head (marked in black circle in Fig. 5(e)). This failure was caused by the low elastic module, which itself was the result of severe osteoporosis (Table 2). The osteoporosis bone elements are much more vulnerable than normal bone elements.
4. The final shape of the umbrella is almost identical in Scenario 2 and 4, which means that the initial position of the sleeve does not affect the final shape of umbrella. However, the traces left by the sleeve are much different. In Scenario 2, when the sleeve is placed at the bottom and the temperature is rising, the sleeve expanded and dragged by the umbrella-shaped support device. A columnar hole of 20 mm diameter and 22.5 mm depth is formed along the decompression channel (Fig. 5(d)). However, in Scenario 4, the sleeve was placed in the middle and there was no additional damage done to the decompression channel (Fig. 5(f)).
5. There are stresses placed upon the support device and formal head because of the shape taken during the recovery process (Fig. 5(g) and (h)). These stresses correspond to the strains caused by the deformation of the umbrella-shaped device. The cancellous bone inside the femoral bone partially prevents the device from recovering to its original shape; thus the device squeezes the femoral head from inside to outside. The stresses and strain are helpful for the formal head to support the pressure brought upon the femoral head during daily activities. This means the umbrella-shaped support device is much more effective than other devices. The usefulness of this feature can be seen in the porous tantalum rod implant [21]: when there is almost no deformation in the implant device, additional enforcement of the femur head is only created by the implant device’s stiffness.

## Discussion

The implant surgery process includes opening the umbrella-shaped device. This device cuts through the cancellous bone and soft tissue inside the femoral head, thus causing element failure. The explicit integration method in Lsdyna is used to simulate the implant process.

In this study, a 3-step scheme is used to construct the formal head FEA mesh model. The Correspond Method is used for assigning material to the mixed-element type mesh which is not suitable for auto-assigning material in Mimics. Finally, based on Lsdyna, an analysis is conducted with a dynamic relaxation for the stress initialization wherein the prescribed geometry is obtained from the pre-step.

The simulation revealed that inside the formal head the final shape of the umbrella-shaped device is not the same as the original shape. An outward expansion strain is formed inside the formal head because of the resistance created during the recovery process of the device. Penetration traces corresponding to the umbrella arms were formed inside the formal head. The traces were not strictly made along the radial direction, with some deflection caused by the uneven bone density.

The results of the analysis indicate that the simulation method is suitable and accurate. More specifically, the maximum radius of the simulation’s umbrella-shaped device was almost identical to the observation data. Placing the sleeve in the middle (position II) is a better choice than the bottom of the umbrella (position I), because there is no additional damage caused in this position to the decompression channel.

Interestingly, during the simulation we noted that if the patient suffered from severe osteoporosis, and then the operation would fail. More specifically, the failure occurred when the umbrella arm punctured the formal head. In other words, a bone mineral density test will help a doctor determine if a patient is suitable for the implant surgery.

The proposed FEA method for a computational study on the surgery simulation is proven to be reasonable and effective. For an individual patient, different sizes of support devices, initial positions and other conditions can be simulated. In other words, an individual treatment for different patients is possible with pre-FEA for specialized treatment. Our later study will focus on the evaluation of the effect of the Ni-Ti implant treatment by comparing the bearing capacity of the formal head before and after surgery through FEA.
